# Characterization of the preferred cation cofactors of chloroplast protein kinases in *Arabidopsis thaliana*

**DOI:** 10.1002/2211-5463.13563

**Published:** 2023-01-31

**Authors:** Roberto Espinoza‐Corral, Serena Schwenkert, Anja Schneider

**Affiliations:** ^1^ Department of Biochemistry and Molecular Biology Michigan State University East Lansing MI USA; ^2^ Plant Molecular Biology, Faculty of Biology Ludwig Maximilians University Munich Planegg Germany

**Keywords:** *Arabidopsis*, cation homeostasis, chloroplast, protein phosphorylation

## Abstract

Chloroplasts sense a variety of stimuli triggering several acclimation responses. One prominent response is the mechanism of state transitions, which enables rapid adaption to changes in illumination. Here, we investigated the link between divalent cations (calcium, magnesium, and manganese) and protein kinase activity in *Arabidopsis* chloroplasts. Our results show that manganese ions are the strongest activator of kinase activity in chloroplasts followed by magnesium ions, whereas calcium ions are not able to induce kinase activity. Additionally, the phosphorylation of specific protein bands is strongly reduced in chloroplasts of a cmt1 mutant, which is impaired in manganese import into chloroplasts, as compared to the wild‐type. These findings provide insights for the future characterization of chloroplast protein kinase activity and potential target proteins.

## Graphical abstract

Plant chloroplasts contain protein kinases that regulate vital processes such as photosynthesis. Despite their similarity with other kinases of eukaryotic origin, chloroplast kinases have shown features that suggest their divergence from the eukaryotic branch, such as their preference for cation cofactors. Using chloroplasts from the model plant *Arabidopsis thaliana*, we demonstrate that chloroplast kinases prefer manganese and magnesium over calcium.

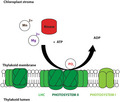

**Abbreviations**
ABC1K: absence of bc1 complex kinase
Ca: calcium
CMT1: chloroplast manganese transporter 1
CSK: chloroplast sensor kinase
Mg: magnesium
Mn: manganese.
pCKII: plastid casein kinase II
PQ: plastoquinone
PS: photosystem

Over the past four decades, chloroplasts have been reported to sense environmental cues triggering orchestrated responses, which updated their role in plant stress adaptation. The responses triggered in chloroplasts can impact processes such as gene expression and lipid biosynthesis under temperature fluctuations [1, 2, 3, 4], immune responses with the synthesis of phytohormones [5], and light harvesting by photosystem I (PSI) and photosystem II (PSII) as a result of changes in light quality known as state transitions [6, 7, 8, 9]. Interestingly, the regulatory mechanism of state transitions involves protein phosphorylation [10, 11], which is predominantly mediated by the chloroplast kinases STN7 and STN8 [12, 13]. Furthermore, proteins related to photosynthesis are not the only ones reported to undergo phosphorylation [14, 15], which suggests that this post‐translational modification could govern multiple processes in chloroplasts.

Several surveys of yet uncharacterized chloroplast protein kinases suggest that, unlike well‐known protein kinases from the eukaryote protein kinase (ePK) family, plastid protein kinases could belong to the atypical protein kinase (aPK) family found in prokaryotes [16, 17, 18, 19]. The chloroplast protein kinases characterized so far are only a few examples, that is, STN7/STN8, members of the Absence of bc_1_ Complex kinase (ABC1K) family [20, 21, 22], plastid casein kinase II (pCKII) [23] and the chloroplast sensor kinase (CSK) [24]. Interestingly, experimental evidence suggests that some of these protein kinases prefer manganese over other di‐valent cations as a cofactor [24, 25]. This poses the question on the overall requirement of manganese ions for their activity. Therefore, it is still unclear whether chloroplasts’ protein kinases require calcium as cofactor like ePK or perhaps prefer other di‐valent cations present in chloroplasts such as manganese and magnesium.

Here we investigated the role of calcium (Ca), magnesium (Mg), and manganese (Mn) ions as cofactors for chloroplast protein kinases *in organello* using phosphorylation assays in *Arabidopsis* chloroplasts. Our results suggest that chloroplast protein kinases are active in the presence of Mg and Mn but inactive when only Ca is used as cofactor. Furthermore, using chloroplasts from the knockout mutant *cmt1* [26, 27], which possess strongly reduced Mn contents in the chloroplast [26, 27], we observed that the protein phosphorylation pattern in the mutant chloroplast is strongly reduced at two specific molecular weights, supporting the idea that chloroplasts contain Mn‐dependent protein kinases. Collectively, our results highlight the importance of Mn ions as the preferred cofactor for the overall protein kinase activity in chloroplasts followed by Mg ions. We also provide a list of putative protein targets for the activity of Mn‐dependent kinases that could be tested in future experiments.

## Materials and methods

### Chloroplast isolation

Three weeks old *Arabidopsis thaliana* wild‐type (Col‐0 ecotype) or *cmt1* (SALK_129037c, grown for 6 weeks) plants were grown onto soil under 12 h light and 12 h dark periods (120 μmol photons s^−1^·m^−2^ light intensity).

When isolating intact chloroplasts, the foliar tissue was ground in a kitchen blender with Isolation buffer (330 mm sorbitol, 50 mm HEPES pH 7.5, 2 mm EDTA, 1 mm MgCl_2_, 5 mm ascorbic acid) and filtered through one layer of gauze. This solution was centrifuged for 5 min at 1500 ***g*** and 4 °C to rescue the pellet and resuspend it in Washing buffer (330 mm sorbitol, 50 mm HEPES pH 7.5), which was loaded on percoll gradients of 40% percoll solution (330 mm sorbitol, 50 mm HEPES pH 7.6, 40% percoll) and 80% percoll solution (330 mm sorbitol, 50 mm HEPES pH 7.6, 80% percoll) and centrifuged for 5 min at 8000 ***g*** and 4 °C and the intact chloroplasts rescued to be washed with washing buffer.

For the separation of chloroplast into stroma and membrane fractions, the procedure skipped the percoll gradient and continued with resuspension of crude chloroplasts in osmotic shock buffer (0.6 m sucrose, 1 mm EDTA, 10 mm Tricine pH 7.9). The sample was then incubated on ice for 30 min under darkness and centrifuged for 1 h at 100 000 ***g*** and 4 °C to separate the soluble fraction (stroma) from the pellet (membranes).

### *In‐vitro* kinase assay

Chloroplast samples were incubated with 3 μCi of [γ‐^32^P] ATP in 40 μL kinase reaction buffer containing 20 mm Tris pH 7 and the given di‐valent cation (Mg, Mn, or Ca chloride). The reaction was carried out for 30 min at room temperature and stopped by adding 5 μL of SDS loading buffer without boiling the samples.

### SDS/PAGE electrophoresis and autoradiography exposure

Radiolabeled samples containing SDS loading buffer were separated on homemade 12% SDS/polyacrylamide gels [28] and stained with Coomassie brilliant blue. After distaining the gels and incubating them in water, gels were vacuum dried and exposed to autoradiography films for 2 h at room temperature.

### Protein and chlorophyll quantification

Protein content was measured using BCA method (Pierce BCA Protein Assay Kit, 23227, Thermo Scientific) and chlorophyll content was measured after acetone extraction as described by Arnon [29].

### Mass spectrometry analyses

Excised coomassie‐stained gel pieces were washed three times with LC–MS grade water and cut into smaller pieces (< 1 mm). Samples were washed twice for 10 min with 20 mm ammonium bicarbonate (ABC) and 100 μL acetonitrile (ACN), respectively. To reduce disulfide bridges, 100 μL 10 mm dithiothreitol (in 20 mm ABC) was added to the sample and incubated at 56 °C for 30 min. Then, the samples were washed with 100 μL ACN for 10 min and 100 μL 55 mm iodoacetamide (in 20 mm ABC) for 30 min in the dark. Afterwards, the samples were washed for 2 × 10 min with ABC and ACN separately and digested overnight with 0.1 μg·μL^−1^ trypsin (Serva) at 37 °C. Resulting peptides were desalted with homemade C18 stage tips [30], vacuum dried, and stored at −80 °C.

Liquid chromatography–tandem mass spectrometry (LC–MS/MS) was performed on a nano‐LC system (Ultimate 3000 RSLC, ThermoFisher Scientific, Waltham, MA, USA) coupled to an Impact II high‐resolution Q‐TOF (Bruker Daltonics, Bremen, Germany) using a CaptiveSpray nanoelectrospray ionization (ESI) source (Bruker Daltonics). The nano‐LC system was equipped with an Acclaim Pepmap nano‐trap column (C18, 100 Å, 100 μm × 2 cm) and an Acclaim Pepmap RSLC analytical column (C18, 100 Å, 75 μm × 50 cm), both from ThermoFisher Scientific. The peptide mixture was separated over a 40‐min linear gradient of 5–37% (v/v) ACN at a constant flow rate of 250 nL·min^−1^. The column was kept at 50 °C in a column oven throughout the run. MS1 spectra with a mass range from *m*/*z* 200–2000 were acquired at 3 Hz, and the 18 most intense peaks were selected for MS/MS analysis using an intensity‐dependent spectrum acquisition rate of between 4 and 16 Hz. Dynamic exclusion duration was 0.5 min.

Raw files were processed using the maxquant software 2.1.3.0 [31]. Peak lists were searched against the *Arabidopsis* reference proteome (Uniprot, www.uniprot.org, version March 2022) using the built‐in Andromeda search engine with default settings. Proteins were quantified across samples using the label‐free quantification algorithm [32]. Downstream statistical analysis was performed using perseus version 2.0.0 [33] and graph pad prism version 9.4.1. All biological replicates were grouped and log_2_ LFQ intensities were filtered to contain valid values in at least three of the four replicates in at least one group. To enable statistical evaluation, missing values were imputed with random numbers drawn from a normal distribution. A student *t*‐test as implemented in the Perseus was performed. Protein groups with a fold change relative to the wild‐type larger than 1.5 (up‐regulated) or lower than −1.5 (down‐regulated) and with an FDR < 0.05 were considered to show a significant change. Proteins groups were further filtered according to the molecular weight of the gel slices considering a range of ± 5 kDa.

The mass spectrometry proteomics data have been deposited to the ProteomeXchange Consortium via the PRIDE partner repository with the dataset identifier PXD037286.

## Results

### Assessment of the chloroplast protein kinase activity under different buffer conditions.

**Fig. 1:**
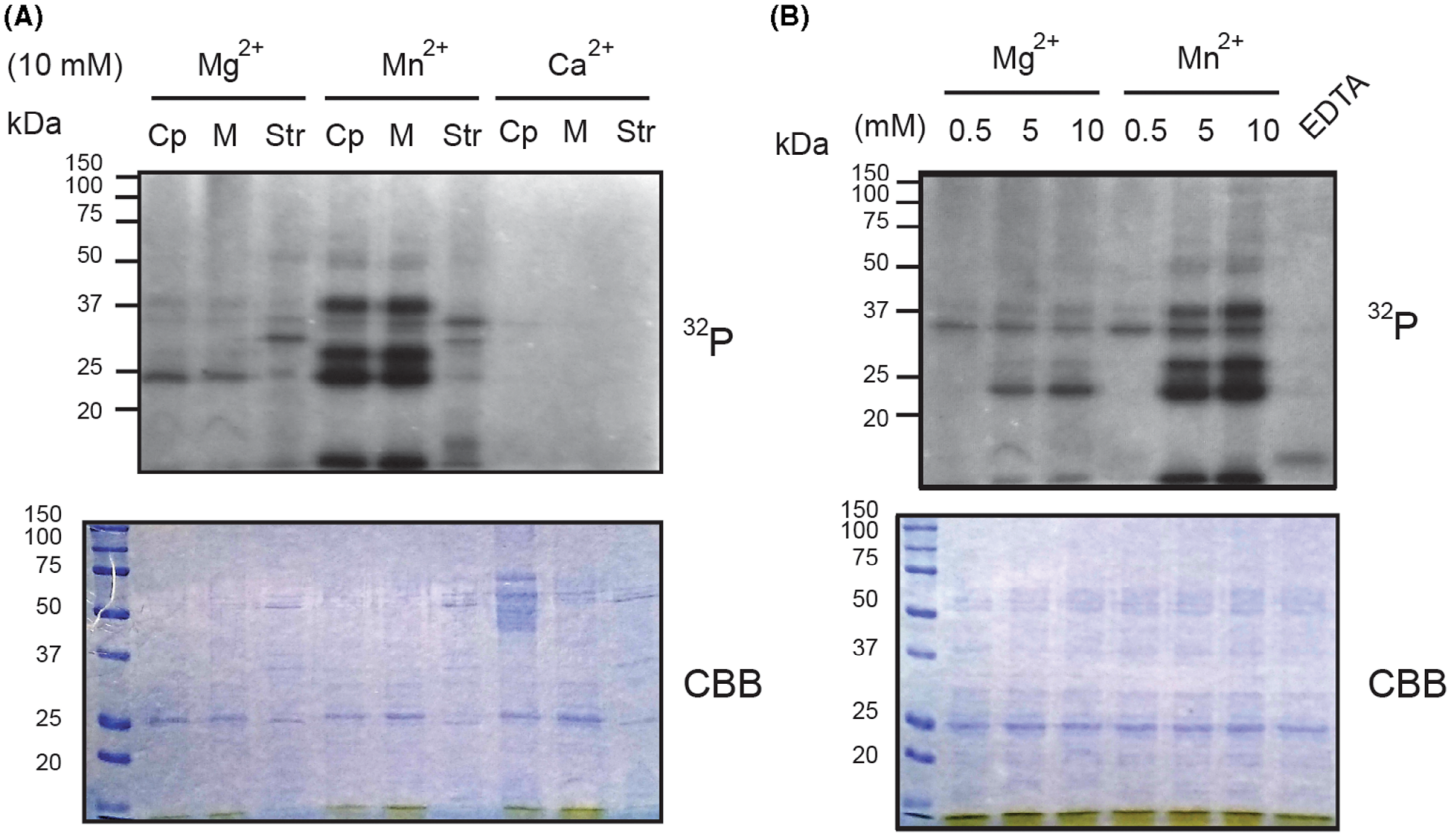
Chloroplast kinase activity using different di‐valent cation cofactors. Wild‐type *Arabidopsis* chloroplasts (Cp) were isolated and separated into their soluble stroma (Str) and membrane (M) fraction. The kinase activity for each fraction was assessed by *in‐vitro* kinase assays. (A) The kinase activity of the soluble and membrane fractions of chloroplasts were tested using magnesium, manganese, or calcium as cofactors. Each sample contained 4 μg of protein. (B) Isolated chloroplasts were broken by osmotic stress and incubated with radiolabeled ATP under increasing concentrations of magnesium and manganese chloride. As a negative control for kinase activity, a sample containing manganese and magnesium chloride was supplemented with 20 mm EDTA. Each sample contained 1 μg of chlorophyll.

Chloroplast protein kinases have been found associated with the thylakoid membrane (STN7/STN8 and ABC1K in plastoglobules lipid droplets) and in the soluble stroma fraction (CSK and pCKII). To test the kinase activity of these sub‐compartments separately, we isolated chloroplasts from *Arabidopsis* wild‐type plants and isolated stroma and membrane fractions. First, we tested the role of three different di‐valent cations as cofactor for protein phosphorylation, while maintaining the buffer conditions. Surprisingly, when Mn ions were added as cation cofactor, we obtained the strongest protein phosphorylation signal for stroma and membrane fractions (Fig. 1A). The protein phosphorylation signal was less when Mg ions were added as cofactor and no kinase activity signal was detected when Ca ions in equal concentrations were added as cofactor. These results suggest a clear preference for Mn ions followed by Mg ions as cation cofactor for chloroplast protein kinase activity. Furthermore, when comparing the stroma fractions of Mg and Mn ions treatments, the signal intensity of two bands around 37 kDa changed. The signal intensity of the upper band was more pronounced, when Mn ions were added, whereas the signal intensity of the lower band was stronger when Mg ions were added. Moreover, the signal intensity of the membrane proteins was much stronger when Mn ions were used as cofactor, despite a similar pattern of protein phosphorylation compared when Mg ions were added.

To further corroborate the preference for Mn ions and Mg ions by total chloroplast kinase activity, we assessed protein phosphorylation under titrated concentrations of both cations. Indeed, the protein phosphorylation increases with higher cation concentration (Fig. 1B). Interestingly, the kinase activity is close to its maximum intensity at 5 mm for both cations. As a negative control for the effect of the cofactors, EDTA, as a chelating agent, was added to chloroplast samples and treated with 10 mm of Mg and Mn chloride.

### Comparison of chloroplast kinase activity in Mn‐deprived chloroplasts.

**Fig. 2:**
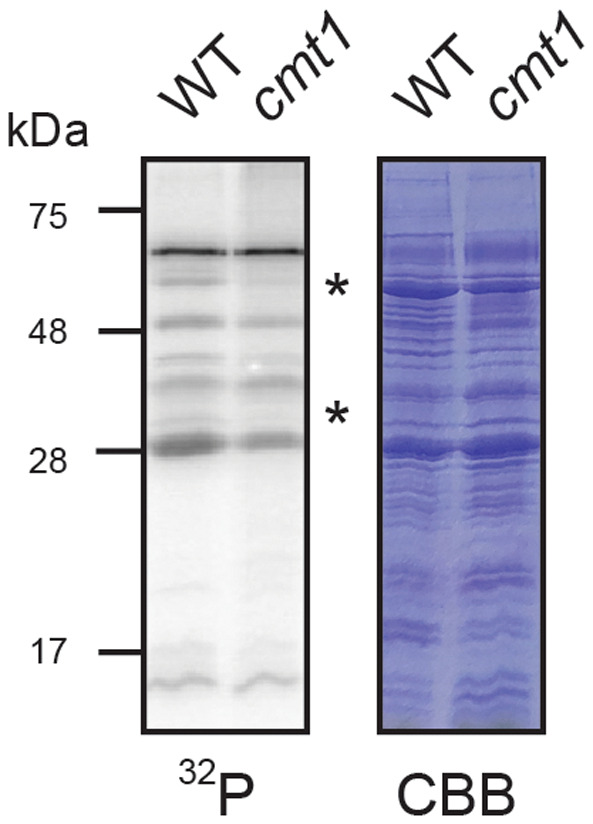
Comparison of the kinase activity from *cmt1* mutant chloroplasts. Intact chloroplasts isolated from wild‐type and *cmt1* mutant plants were incubated with radiolabeled ATP in the presence of 5 mm manganese chloride to subsequently visualize their protein phosphorylation pattern. The protein bands that are strongly reduced in the *cmt1* chloroplasts sample compared with wild‐type are indicated with asterisk. These bands were excised afterwards from a nonradioactive gel and subjected to mass spectrometry analyses.

**Fig. 3:**
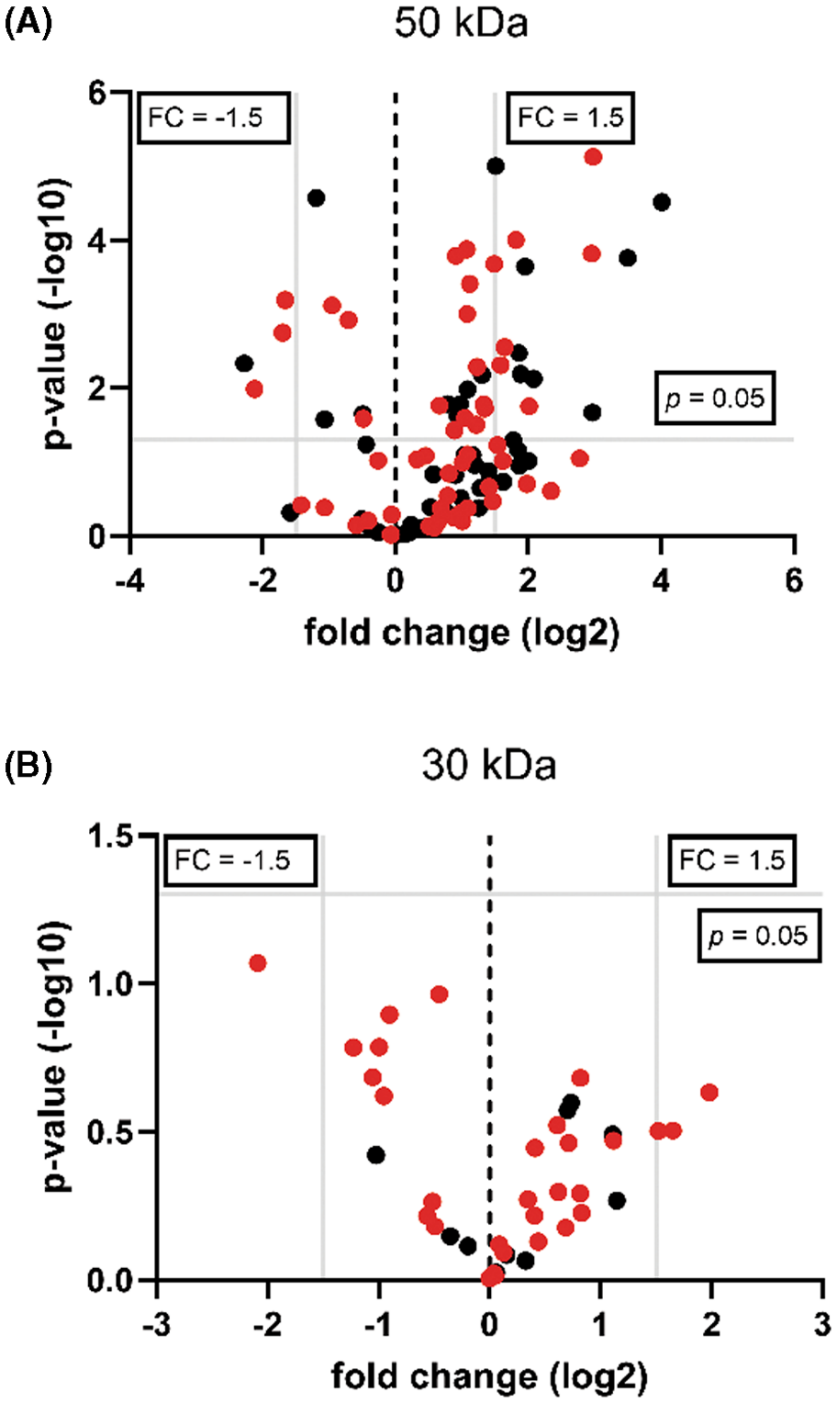
Volcano plot of the protein groups identified by mass spectrometry. The plots show the proteins found in the excised SDS gel bands at 50 kD (A) and 30 kD from wild‐type and *cmt1* chloroplasts (B). Red points represent potentially phosphorylated proteins. Gray lines indicate *P* value = 0.05 and fold change ± 1.5. *n* = 4.

Since the strongest signal of protein phosphorylation was obtained using Mn ions as cofactor, we hypothesized that chloroplasts with reduced Mn contents, as described for the *cmt1* mutant, should have weaker kinase activity compared with wild‐type. For this purpose, we isolated intact chloroplasts from *Arabidopsis* wild‐type plants and *cmt1* plants [26] to compare their kinase activity in phosphorylation assays. Interestingly, two protein phosphorylation bands were strongly reduced in *cmt1* chloroplasts with molecular masses of around 30 and 50 kDa, which might indicate that Mn ions are required for the activity of specific protein kinases (Fig. 2). Furthermore, the proteins present around 30 and 50 kDa represent potential targets for putative Mn‐dependent protein kinases that we attempted to identify by subjecting gel slices excised from four replicates of wild‐type and *cmt1* chloroplasts at 30 kDa and 50 kDa to mass spectrometry. To rule out the possibility that the reduction in protein phosphorylation is the result of differences in protein abundance between wild‐type and *cmt1* chloroplasts, we quantified protein abundance by label‐free quantification [32] (Tables S1 and S2). The identified proteins after filtering resulted in 98 proteins identified in the 50 kDa band and 38 proteins identified in the 30 kDa band (Table S3). Interestingly, 55 of the 98 proteins found in gel slices around 50 kDa have been reported to be phosphorylated [34, 35, 36, 37] with 28 of them not showing statistically significant differences in protein abundance as verified by a student *t*‐test and shown in a volcano plot (Fig. 3A, Table S3). Moreover, only three of the potential target proteins were reduced by a higher log_2_ fold change than 1.5. Even more striking, for the 30 kDa band 30 of the 38 proteins filtered in slices at 30 kDa were reported to undergo phosphorylation with none of them having differences in protein abundance comparing wild‐type and *cmt1* (Fig. 3B, Table S3). Further filtering for proteins with high abundance and showing no significant difference between wild‐type and *cmt1*, we found 5 putative targets for each gel slice at 50 and 30 kDa, respectively (Table 1). We hypothesize that these proteins represent potential targets for the activity of Mn ions dependent kinases in *Arabidopsis* chloroplasts that could be tested in future experiments.

**Table 1:** Putative protein targets for Mn‐dependent protein kinase activity. Proteins are ordered according to their abundance in each gel slice. Proteins found to undergo phosphorylation are reported according to Sugiyama *et al.* [36]^a^, Xi *et al.* [37]^b^, Lohring *et al.* [35]^c^, Reiland *et al.* [34]^d^.

Primary accession n°	Protein name
Gel slices at 50 kDa	
AT1G42970^a,b^	Glyceraldehyde‐3‐phosphate dehydrogenase GAPB
AT3G48730^c,b^	Glutamate‐1‐semialdehyde 2,1‐aminomutase 2
AT4G18480^d,b^	Mg‐chelatase subunit ChlI‐1
AT3G56940^b^	Mg‐protoporphyrin IX monomethyl ester [oxidative] cyclase
AT5G54810^b^	Tryptophan synthase beta chain 1
Gel slices at 30 kDa	
AT2G35410^b,d^	RNA‐binding protein CP33B
AT5G24490^b,d^	30 S ribosomal protein
AT2G34460^b^	Uncharacterized protein At2g34460
AT1G65260^b^	Membrane‐associated protein VIPP1
AT4G04020^b,d^	Fibrillin 1a

## Discussion

Protein phosphorylation has proven to be an important mechanism to regulate processes that require reversible and rapid responses specially under stress conditions. In chloroplasts, the most well‐described mechanism is state transitions where the protein kinases STN7/STN8 become more active when sensing a reduced plastoquinone (PQ) pool. STN8 mainly phosphorylates core proteins of PSII (for photo‐repair) and STN7 the light‐harvesting complexes, which enables LHCII to move towards PSI to avoid saturation of the electron transport chain [38, 39, 40]. Likewise, CSK has been shown to sense the PQ pool as well, becoming active under oxidizing conditions repressing the expression of genes encoding for PSI proteins [41]. Moreover, members of the ABC1K family have been shown to respond to light showing reduced kinase activity under high light stress [22] and preference for Mg and Mn ions as cofactors [25].

The activity of protein kinases in chloroplasts has been investigated in *in‐vitro* assays using radiolabeled ATP and different buffer conditions. Interestingly, a particular buffer composition was required for the CSK and ABC1K protein kinases, which suggests that chloroplasts kinases might differ from the well‐known cytosolic kinases that use Ca ions as cofactor [25, 41]. Indeed, our results using *Arabidopsis* wild‐type chloroplasts indicated a clear preference for Mg ions and Mn ions instead of Ca ions. Moreover, Mn ions activated more strongly the kinase activity than that of Mg ions (Fig. 1). Surprisingly, when Ca ions were added to membranes or stroma fractions no protein phosphorylation was observed. These results show that protein kinases in chloroplast might have evolved separately from other kinases that prefer Ca ions as cofactor [42, 43]. In addition to our observations on Mn‐dependent activity of protein kinases in chloroplasts [25], there are several other proteins that require Mn ions as cofactor. Mn is a vital component of the oxygen‐evolving‐complex of PSII [44, 45, 46] and Mn ions are required as cofactors for enzymatic activities, like the thylakoid associated phosphatase of 38 kDa [46, 47], 3‐deoxy‐d‐arabino‐heptulosonate 7‐phosphate synthase [48] and Imidazoleglycerol‐phosphate dehydratase [49].

In order to analyze kinase activity in natural Mn‐deficient chloroplasts, we made use of the *cmt1* mutant. The *cmt1* mutant is severely compromised in growth and photosynthetic activity, and chloroplasts possess only 30% Mn content in comparison with wild‐type [26, 27]. In our experiments, the kinase activity of chloroplasts from both wild‐type and *cmt1* were similar in pattern and intensity, and moreover, kinase activity in both lines was identical in the presence of exogenous Mn (Fig. 2) and in the absence of exogenous Mn (Fig. S1). These findings indicate that approximately 30% Mn content in chloroplasts is sufficient to maintain most of the protein kinase activity. Although we cannot rule out pleiotropic effects in *cmt1* mutant, only two bands around 50 and 30 kDa showed a strong reduction of protein phosphorylation in chloroplasts from *cmt1* compared with wild‐type. In case of a pleiotropic effect, we would have expected a general decline in protein phosphorylation. A closer inspection of the most abundant proteins around 50 and 30 kDa, that have been reported to undergo phosphorylation, revealed several putative substrate candidates for Mn‐dependent kinase activity. At 50 kDa, glyceraldehyde‐3‐phosphate dehydrogenase (involved in the Calvin and Benson cycle) and glutamate‐1‐semialdehyde 2,1‐aminomutase 2 were identified (Table 1). Putative substrate candidates at 30 kDa were chloroplasts ribosomal proteins and the RNA‐binding protein CP33B (Table 1) known to bind specifically to *psbA* mRNA [50]. It is tempting to speculate that an Mn ion‐dependent kinase phosphorylates CP33B thereby exerting a regulatory role in *psbA* translation. Interestingly, the amount of *psbA* mRNA is reduced to 50% in *cmt1* plants in comparison with wild‐type [26].

Taken together, our results suggest that most chloroplast protein kinases can accommodate Mg ions or Mn ions as cofactors. There could be some protein kinases, which might require specifically Mn ions as cofactor as shown by the reduction of protein phosphorylation in *cmt1* mutant chloroplasts. It will be interesting to identify those protein kinases in future studies.

## Conflict of interest

The authors declare no conflict of interest.

## Author contributions

REC, SS, and AS designed experiments; REC and SS performed the experiments and analyzed the data; REC wrote the manuscript; SS and AS made manuscript revisions.

## Supporting information


**Fig. S1.** Comparison of the kinase activity from *cmt1* mutant and wild‐type chloroplasts.

**Table S1.** Mass spectrometry results from the band excised at 50 kDa.
**Table S2.** Mass spectrometry results from the band excised at 30 kDa.
**Table S3.** Filtered data according to the sizes of the gel slices.

## Data Availability

The data that support the findings of this study are openly available in PRIDE repository at https://www.ebi.ac.uk/pride/, following reviewer's credentials: Project Name: Surveying the chloroplast protein kinase preferences for cation cofactors in *Arabidopsis thaliana*. Username: reviewer_pxd037286@ebi.ac.uk Password: oHuc9Gra.
