# Supplementary figures and images for: Characterization of the preferred cation cofactors of chloroplast protein kinases in Arabidopsis thaliana
Source: FEBS Open Bio. 2023 Jan 31;13(3):511–8. doi: 10.1002/2211-5463.13563 (PMC9989932; doi:10.1002/2211-5463.13563)

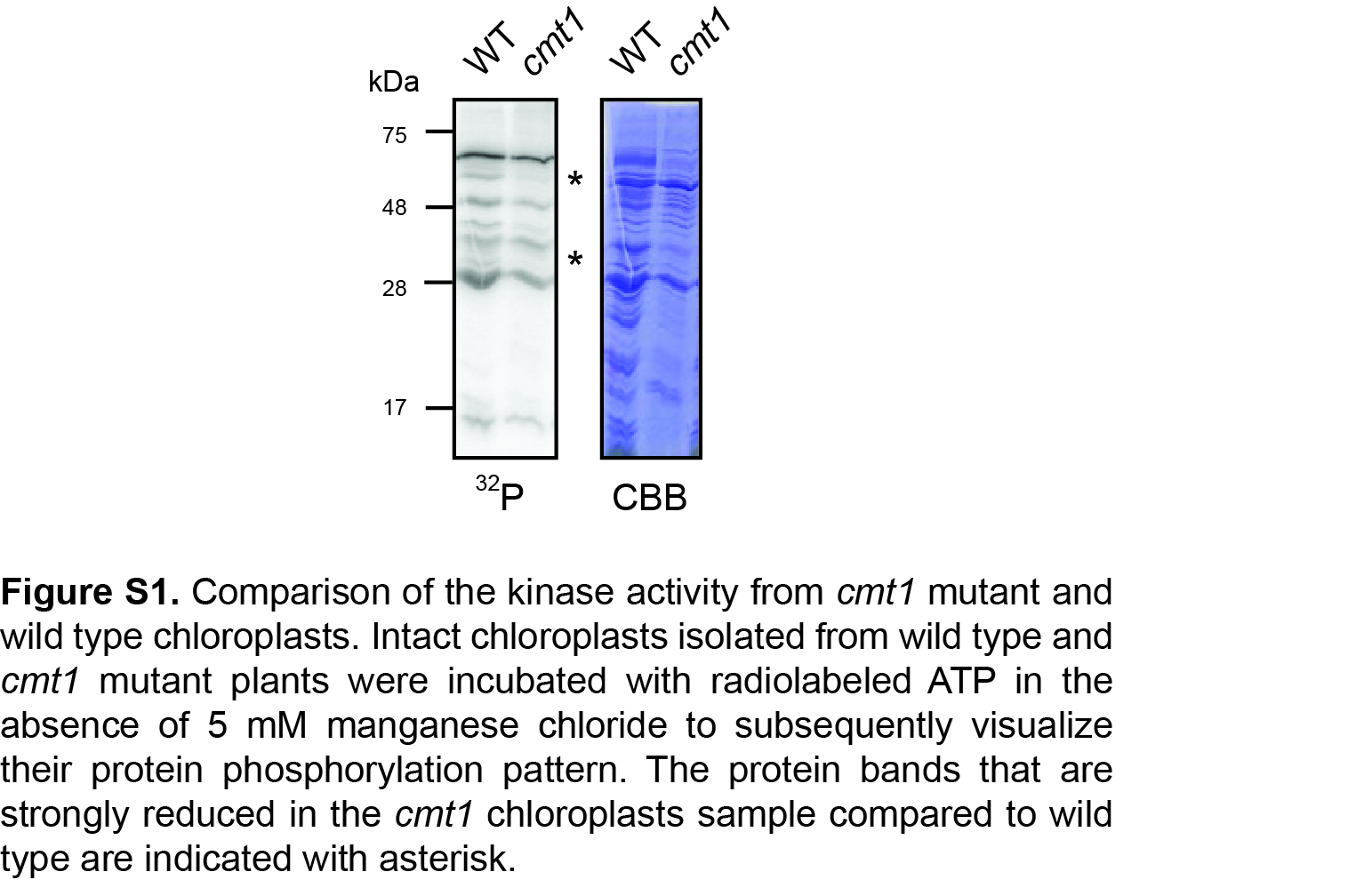

Supplement: Supplementary file 1 — Fig. S1. Comparison of the kinase activity from cmt1 mutant and wild‐type chloroplasts. [file FEB4-13-511-s002.tif]
